# Soluble and plaque amyloid associations with peripheral glucose dysregulation modulated by tau pathology in Alzheimer’s disease

**DOI:** 10.1016/j.tjpad.2025.100459

**Published:** 2026-01-01

**Authors:** Dong Woo Kang, Suhyung Kim, Sunghwan Kim, Yoo Hyun Um, Sheng-Min Wang, Seunggyun Ha, Sonya Youngju Park, Seung-Hwan Lee, Yeong Sim Choe, Donghyeon Kim, Chang Uk Lee, Hyun Kook Lim

**Affiliations:** a Department of Psychiatry, Seoul St. Mary’s Hospital, College of Medicine, The Catholic University of Korea, Seoul, Republic of Korea; b Department of Psychiatry, St. Vincent’s Hospital, College of Medicine, The Catholic University of Korea, Seoul, Republic of Korea; c Department of Psychiatry, Yeouido St. Mary’s Hospital, College of Medicine, The Catholic University of Korea, Seoul, Republic of Korea; d Division of Nuclear Medicine, Department of Radiology, Seoul St. Mary's Hospital, College of Medicine, The Catholic University of Korea, Seoul, Republic of Korea; e Division of Nuclear Medicine, Department of Radiology, Yeouido St. Mary’s Hospital, College of Medicine, The Catholic University of Korea, Seoul, Republic of Korea; f Division of Endocrinology and Metabolism, Department of Internal Medicine, Seoul St.Mary's Hospital, The Catholic University of Korea, Republic of Korea; g Research Institute, NEUROPHET Inc., Seoul 06247, Republic of Korea; h CMC Institute for Basic Medical Science, the Catholic Medical Center of The Catholic University of Korea, Seoul, Republic of Korea

**Keywords:** Alzheimer disease, Amyloid beta-peptides, Tau proteins, Diabetes mellitus, Type 2, Positron-emission tomography

## Abstract

**Background:**

Glucose metabolic dysfunction in Alzheimer’s disease (AD) has been reported to be associated with soluble amyloid-β oligomers (OAβ) and plaque amyloid. However, the potential modulatory role of tau pathology in these associations remains to be fully elucidated.

**Objectives:**

To investigate whether tau pathology modifies the relationship between plasma OAβ burden, plaque amyloid, and systemic glucose metabolism in individuals across the AD spectrum.

**Design:**

Cross-sectional observational study.

**Setting:**

Memory clinic-based cohort from a single tertiary academic medical center in Republic of Korea.

**Participants:**

A total of 113 older adults, including cognitively normal individuals, patients with mild cognitive impairment, and Aβ-PET–positive dementia patients.

**Measurements:**

Plasma oligomeric Aβ (OAβ) levels were measured in blood samples using the Multimer Detection System, which quantifies oligomeric forms of Aβ in plasma. Aβ plaque deposition was assessed using [^18^F]-flutemetamol PET, and tau pathology was assessed using [^18^F]-flortaucipir PET, from which Braak staging was determined. Glucose metabolism was evaluated using fasting plasma glucose and hemoglobin A1c (HbA1c). Generalized linear models were used to examine the associations and potential interactions between plasma OAβ burden and plaque Aβ with tau pathology, adjusting for clinical covariates.

**Results:**

A significant interaction was identified between plasma OAβ levels and Braak stage III/IV, but not Braak I or V/VI, when referenced to Braak 0. Only at Braak 0, higher plasma OAβ levels were associated with higher HbA1c compared with Braak stage III/IV (β = −4.191, 95 % CI −7.714 to −0.669, *p* = 0.020). No significant interactions were observed for fasting glucose or for Aβ-PET SUVR. Sensitivity analyses adjusting for diabetes diagnosis and excluding dementia participants confirmed the robustness of these findings.

**Conclusion:**

Soluble Aβ oligomers, rather than plaque amyloid, are selectively associated with systemic glucose dysregulation in the absence of overt tau pathology. Tau staging may be crucial for identifying AD subgroups vulnerable to metabolic dysfunction potentially associated with early Aβ toxicity.

## 1 Introduction

Growing evidence suggests that Alzheimer’s disease (AD) may involve mechanisms similar to those observed in Type 2 diabetes mellitus (T2DM), leading to the conceptualization of AD as a disorder of brain insulin resistance [1]. In this context, systemic metabolic dysregulation, characterized by peripheral insulin resistance and chronic hyperglycemia, is thought to impair central insulin signaling [1]. These disturbances compromise insulin transport across the blood–brain barrier, activate proinflammatory pathways, and promote the accumulation of advanced glycation end-products, collectively disrupting neuronal glucose metabolism [2]. Such metabolic dysfunction accelerates amyloid-β (Aβ) deposition and tau hyperphosphorylation, ultimately contributing to neurodegeneration [[3], [4], [5]]. This framework establishes a mechanistic link between systemic metabolic disorders and AD pathology, highlighting the pivotal role of impaired insulin signaling in disease progression.

Among the pathological hallmarks of AD, oligomeric Aβ (OAβ) has emerged as a key mediator of neurodegeneration linked to metabolic dysfunction, exerting toxic effects that exceed those of its monomeric form [6]. OAβ disrupts insulin signaling by inhibiting insulin receptor autophosphorylation, impairing the activation of downstream intracellular enzymes, and reducing glucose transporter 4 translocation to the membrane [6]. It also impairs synaptic function by disrupting long-term potentiation and activating inflammatory pathways, including TNF-α and PKR signaling, further exacerbating insulin resistance [7,8]. Moreover, OAβ affects peripheral glucose homeostasis by disrupting hypothalamic regulatory pathways, thereby promoting central insulin resistance [9].

OAβ levels are measured in plasma using the Multimer Detection System (MDS-OAβ), which captures oligomer-specific epitopes to distinguish oligomers from monomers and fibrillar forms [10]. This blood-based assay provides a minimally invasive approach to detect early, dynamic changes in Aβ pathology that often precede plaque formation observed on amyloid positron emission tomography (PET) [[11], [12], [13]], offering complementary information on soluble, neurotoxic species linked to synaptic dysfunction and tau phosphorylation [8,14]. Although amyloid-PET reliably measures fibrillar amyloid burden, it does not detect soluble oligomers that may more directly reflect neurotoxicity and metabolic dysfunction, highlighting the added value of plasma oligomeric Aβ measurement in the AD biomarker framework.

Recent findings suggest that OAβ contributes to the initiation of tau pathology through metabolic disturbances linked to insulin resistance. Aβ oligomers accumulate early, peaking before significant tau aggregation, indicating temporal precedence over tau pathology [15]. Mechanistically, OAβ induces tau phosphorylation and inactivates insulin receptor substrate via c-Jun N-terminal kinase activation, while aberrantly activating glycogen synthase kinase-3β, a key mediator of tau hyperphosphorylation [14,16]. These processes connect amyloid pathology with tau-related neurodegeneration through metabolic and signaling pathways that may drive disease progression.

However, clinical findings on the relationship between tau pathology and glucose metabolism have been inconsistent [4]. While some studies have reported associations between insulin resistance and elevated tau levels in cerebrospinal fluid (CSF) [17,18], others using tau PET imaging have failed to demonstrate robust links with glycemic indices [19]. These inconsistencies may partly reflect the fact that prior studies rarely assessed amyloid and tau pathologies in an integrated manner. This underscores the need for a comprehensive evaluation of how glucose dysregulation, Aβ and tau pathologies interact to influence disease progression.

While OAβ is widely regarded as the more neurotoxic species due to its early and direct pathogenic effects [20], amyloid plaques remain the primary surrogate marker of overall Aβ burden in the brain. Most clinical studies examining the relationship between peripheral glucose metabolism and Aβ pathology have focused on amyloid plaques assessed via amyloid positron emission tomography (amyloid-PET). Previous studies reported a significant association between midlife insulin resistance and late-life amyloid plaque deposition [21,22]. However, these studies were primarily conducted in cognitively normal midlife individuals, limiting generalizability across the clinical spectrum of AD. The restricted range of amyloid-PET values in this population may reduce sensitivity to broader metabolic associations. Moreover, a meta-analysis found no significant relationship between glucose metabolism measures and Aβ biomarkers [4], highlighting inconsistencies in the literature. Such discrepancies may reflect variations in covariate adjustment and the lack of consideration for antidiabetic medication use, which directly influences glucose metabolism. Importantly, limited studies have comprehensively assessed both Aβ proteinopathy and tauopathy in relation to glucose metabolism dysregulation, leaving a critical gap in understanding their complex interplay.

Building on this framework, our study aimed to investigate the interplay between OAβ, tau pathology, and glucose metabolism in the context of insulin resistance and metabolic dysfunction. Specifically, we sought to determine whether the relationships of amyloid plaques and tau pathology differ from those of OAβ and tau in their impact on short- and long-term plasma glucose regulation, including fasting blood glucose and HbA1c levels. By directly comparing these distinct amyloid species in conjunction with tau pathology, we aimed to clarify the specific role of OAβ in metabolic dysfunction related to AD. To our knowledge, this is the first study to systematically evaluate these associations, incorporating both soluble and fibrillary amyloid pathology while accounting for core pathological hallmarks of AD.

## 2 Materials and methods

### 2.1 Participants

A total of 113 participants were included in the present analysis, selected from an initial cohort of 373 individuals enrolled in the Catholic Aging Brain Imaging Database (CABID) (Supplementary Fig. S1). Inclusion criteria were: [1] age ≥ 55 years; [2] availability of fasting blood glucose and HbA1c measurements; and [3] availability of MDS-OAβ blood sample, Aβ-PET, and tau-PET scans. Exclusion criteria included clinically significant psychiatric disorders (e.g., major depressive disorder, schizophrenia, bipolar disorder), neurological disorders (e.g., brain tumors, epilepsy), systemic or cerebrovascular diseases affecting cognition, severe sensory impairments, or contraindications for neuroimaging.

The final analytic sample comprised 28 cognitively normal older adults, 59 individuals with mild cognitive impairment (MCI), and 26 patients with AD dementia. All participants had Clinical Dementia Rating (CDR) scores of 0, 0.5, or 1, which corresponded to the range eligible for tau-PET acquisition under the research protocol. Participants with CDR ≥ 1 but negative Aβ-PET scans were excluded in accordance with the protocol. Moreover, individuals with CDR > 1 were not eligible for tau-PET and were excluded due to the potential for reduced medication adherence and increased comorbidity burden, which may confound glucose metabolism assessments.

Cognitively normal individuals were defined as having a global CDR score of 0 and normal performance on the Korean version of the Consortium to Establish a Registry for Alzheimer’s Disease (CERAD-K) battery [23]. MCI was diagnosed based on: [1] subjective memory complaints confirmed by an informant, [2] objective impairment in at least one CERAD-K domain (≥1 SD below age- and education-adjusted norms), [3] preserved functional independence, [4] a global CDR score of 0.5, and [5] absence of dementia according to DSM-V criteria. AD dementia was diagnosed based on the DSM-V and NINCDS-ADRDA criteria for probable AD [24,25], with confirmation of Aβ pathology by positive Aβ-PET and a global CDR score of 1. Neuropsychological testing followed the CERAD-K protocol, with test details provided in the Supplementary Methods.

All clinical, imaging, and laboratory assessments were performed within a three-month baseline period. The study was approved by the Institutional Review Board of Seoul St. Mary’s Hospital, The Catholic University of Korea (IRB No.: SC21TISI0017), and all participants provided written informed consent in accordance with the Declaration of Helsinki.

### 2.2 Measurement of Aβ oligomerization in plasma

Plasma levels of oligomeric amyloid-beta (OAβ) were quantified using the Multimer Detection System for OAβ (MDS-OAβ). Blood samples were collected via venipuncture using ethylene-diamine-tetraacetic acid (EDTA) vacutainer tubes, following established protocols for MDS-OAβ measurement [10]. After collection, EDTA plasma was centrifuged at 3500 rpm for 15 min at room temperature. The resulting plasma was aliquoted into 1.5-mL polypropylene tubes and stored at −70 °C to −80 °C. The samples were then transported to PeopleBio Inc. for MDS-OAβ level analysis.

Prior to measurement, plasma aliquots were thawed at 37 °C for 15 min. MDS-OAβ levels were measured using the multimer detection system, which is CE-marked and approved by the Korean Food and Drug Administration [10,12,13]. To determine amyloid PET positivity and identify amyloid pathology, a cutoff value of 0.78 ng/mL was applied [11]. Furthermore, to classify AD risk, a two-cutoff algorithm was implemented, aligning with methodologies outlined in prior biomarker research [26]. Based on this approach, plasma MDS-OAβ levels were categorized into three risk groups: low risk (<0.78 ng/mL), intermediate risk (0.78–0.93 ng/mL), and high risk (≥0.93 ng/mL). These cutoff values were established in accordance with the Clinical and Laboratory Standards Institute guideline NBS 04-A, which provides a standardized framework for developing and validating reference intervals in laboratory testing.

### 2.3 Assessment of diabetes mellitus diagnosis, antidiabetic medication use, and HbA1c and fasting blood glucose levels

Participants were evaluated for a prior diagnosis of DM and the use of antidiabetic medications in the three months preceding the baseline assessment. T2DM at baseline was defined as meeting one or more of the following criteria: [1] a clinical diagnosis of T2DM, indicated by registration with ICD-10 codes E11–E14; [2] current use of antidiabetic medications, including oral hypoglycemic agents or non-insulin injectable therapies (ATC code A10), excluding insulin therapy; or [3] glycemic criteria consistent with diabetes, defined as fasting plasma glucose (FPG) ≥ 7.0 mmol/L (126 mg/dL), 2-hour plasma glucose (2h-PG) ≥ 11.1 mmol/L (200 mg/dL) after a 75 g oral glucose tolerance test (OGTT), or hemoglobin A1c (HbA1c) ≥ 6.5 % (48 mmol/mol), measured using NGSP/DCCT-aligned and IFCC-standardized assays [27]. Individuals with type 1 diabetes mellitus (ICD-10 code E10) were excluded from the study. Blood-based glycemic markers (FPG, HbA1c) were assessed using standard clinical methods, detailed in the Supplementary Methods.

## 3 Neuroimaging

### 3.1 Image acquisition

All individuals underwent 3D T1-weighted MRI (3T Siemens) scans using a Siemens Skyra 3T scanner with a 20-channel head & neck coil (Siemens Healthcare, Erlangen, Germany) and [^18^F]-flutemetamol and [^18^F]-flortaucipir PET scans with a Biograph 40 TruePoint scanner (Siemens Medical Solutions, Erlangen, Germany). Static PET scans were acquired 90 min after intravenous injection of 185 MBq of [^18^F]-flutemetamol or 80 min after injection of 370 MBq of [^18^F]-flortaucipir for 20 min. The matrix size was 256 × 256 × 175, and the voxel size was 1.3364 × 1.3364 × 3 mm³. Static image reconstruction was performed using the 2D-ordered subsets expectation-maximization (OSEM) algorithm with two iterations for 21 subsets. CT scans were acquired for attenuation correction before the PET scans. Structural T1-weighted images were obtained using the magnetization-prepared rapid gradient echo (MPRAGE) sequence, with the following scanning parameters: repetition time (TR) = 1860 ms, echo time (TE) = 25.3 ms, flip angle = 9°, field of view (FOV) = 224 × 224 mm, matrix size = 256 × 256, 208 axial slices, and a slice thickness of 1.0 mm. All acquired Digital Imaging and Communications in Medicine (DICOM) image files were anonymized and converted to NIfTI format using the “dcm2niix” software.

### 3.2 Image preprocessing

SCALE PET 2.0 (Neurophet Inc., Seoul, Republic of Korea) was used for imaging preprocessing and quantification of [^18^F]-flutemetamol and [^18^F]-flortaucipir PET/CT scans [[28], [29], [30]]. This process involved brain parcellation on coregistered T1-weighted MRI scans. T1-weighted MRIs were corrected for non-uniformity and field distortions before processing. PET images were registered to T1-weighted MRI space, and T1-weighted images were then linearly and non-linearly registered to the Montreal Neurological Institute (MNI) reference space. PET images underwent skull and meninges stripping and were subsequently non-linearly registered to the MNI space using transformations derived from the T1-weighted image to MNI space and PET image to T1-weighted image space. T1-weighted MR images were parcellated into 101 regions using the Desikan-Killiany atlas [31]. No partial volume corrections were applied during the analysis.

### 3.3 Amyloid PET reading & quantification

Amyloid PET images were visually interpreted by assessing regions of interest, including the lateral temporal cortex, frontal cortex, posterior cingulate and precuneus, and inferolateral parietal cortex. Positivity in any of these regions was considered sufficient for an overall positive scan. The final assessment was recorded as a dichotomous classification (positive/negative) based on visual rating, with consensus reached between two nuclear medicine physicians (SH and SYP, both with 14 years of clinical experience in nuclear medicine). Quantitative SUVR values were used as a supplementary measure to assist visual rating, referring to a predefined global SUVR cutoff of 0.62 [32]. The pons was used as the reference region for SUVR calculation in [^18^F]-flutemetamol PET/CT scans studies [33].

### 3.4 Tau PET reading & quantification

Braak staging was utilized for tauopathy staging in the quantitative evaluation of [^18^F]-flortaucipir PET/CT scans, with stages assigned based on anatomical brain regions [34]. Braak I included the entorhinal cortex, while Braak III/IV encompassed the parahippocampal gyrus, fusiform gyrus, lingual gyrus, amygdala, inferior and middle temporal gyri, temporal pole, thalamus, caudal and rostral cingulate, isthmus, posterior cingulate, and insula. Braak V/VI involved the frontal, parietal, and occipital cortex; transverse and superior temporal gyri; precuneus; banks of the superior temporal sulcus; nucleus accumbens; caudate; putamen; precentral and postcentral gyri; paracentral gyrus; cuneus; and pericalcarine [35]. Braak stage II (hippocampus) was excluded from the analysis due to off-target binding of [^18^F]-flortaucipir in this region [36]. In addition, the temporal meta-ROI used for quantitative analysis included the entorhinal cortex, amygdala, parahippocampal gyrus, fusiform gyrus, and the inferior and middle temporal gyri. PET images were displayed according to product information approved by Food and Drug Administration and European Medicines Agency for visual rating. Visual rating was performed regionally for Braak staging, as established by the consensus of two nuclear medicine physicians (SH and SYP, both 14 years clinical experience in nuclear medicine). Quantitative values were referred to for confirmation of staging, comparing of visual rating and SUVR with a pre-established SUVR cutoff of 1.3 [37,38]. If there was a discrepancy between visual rating and quantification-based rating, the visual raters reviewed the images again and made a confirmative rating. This single-cutoff approach was used to complement visual rating to establish a standardized and clinically applicable method for tauopathy staging. Additionally, it has been validated as a prognostic marker for cognitive decline across all disease stages, reinforcing its utility in Tau-PET and in vivo Braak-staging [34]. Braak I positivity was defined as cases where only the Braak I region of interest (ROI) was assessed as positive, while Braak III/IV positivity required the involvement of both Braak I and Braak III/IV ROIs. Braak V/VI positivity was assigned when all Braak ROIs interested (I, III/IV, and V/VI) were involved, whereas Braak 0 indicated that none of the Braak ROIs were assessed as positive. The SUVR for [^18^F]-flortaucipir was calculated using the cerebellar cortex as the reference region to ensure consistency in measurements [34,35].

### 3.5 Apolipoprotein E genotyping

We collected data on apolipoprotein E (*APOE*) genotypes, which may act as confounding factors in the glucose metabolism of older adults [3,21]. *APOE* genotyping was performed using DNA extracted from participants’ peripheral blood. Participants were then classified based on the presence of the *APOE* ε4 allele, with those carrying at least one ε4 allele designated as *APOE* ε4 carriers, while those without any ε4 alleles were classified as non-carriers.

### 3.6 Statistical analysis

Statistical analyses were conducted using R (version 4.3.2) and jamovi (version 2.6.26). To verify previously reported group-level differences in plasma MDS-OAβ levels, initial comparisons were conducted using a subset of 355 participants. Aβ-positive cognitively normal individuals (*N* = 18) were excluded to maintain consistency with prior reports (Supplementary Fig. S1) [13], and analyses were performed across diagnostic groups (Aβ-negative cognitively normal individuals, Aβ-negative MCI, Aβ-positive MCI, and Aβ-positive dementia) using one-way ANOVA.

To examine the distributional characteristics of core AD biomarkers, one-way ANOVA was used to compare plasma MDS-OAβ levels, global Aβ-PET SUVR, and tau-PET SUVR (meta-ROI) across Braak stages (0, I, III/IV, and V/VI). Spearman rank correlations were additionally performed to examine monotonic relationships among core AD biomarkers, including plasma MDS-OAβ, Aβ-PET SUVR, and tau-PET SUVR, in the context of potential non-linearity.

Primary analyses evaluated the associations between plasma MDS-OAβ level or global Aβ-PET SUVR and glycemic indices (HbA1c and fasting glucose), with effect modification by Braak stage. Generalized linear models (GLM) with a Gaussian distribution and identity link were applied, adjusting for age, sex, education, *APOE* ε4 carrier status, antidiabetic medication use, and global CDR score. Interaction terms with Braak stage were included to assess differential effects according to tau pathology severity. Additionally, we compared linear and quadratic parameterizations of MDS-OAβ using likelihood-ratio tests, ΔAIC, and ΔBIC. We retained the quadratic term when it improved fit and when the term or its interaction with Braak stage was statistically significant after covariate adjustment. Parametric inferences were supplemented by Bias-Corrected and Accelerated (BCa) bootstrap 95 % confidence intervals based on 1000 resamples.

To ensure the robustness of parameter estimates against potential violations of normality and homoscedasticity, bias-corrected and accelerated bootstrap confidence intervals based on 1000 resamples were computed for all GLM parameters. In addition, residual diagnostics were conducted using Q–Q plots and residual-versus-fitted value plots (Supplementary Figure S2), which confirmed the adequacy of the Gaussian distributional assumptions for the GLM framework. When significant interactions were identified, simple effect analyses were performed by estimating the associations between amyloid biomarkers and glycemic indices separately within each Braak stage using generalized linear models. This approach enabled evaluation of the conditional effects of amyloid pathology across distinct levels of tau burden.

To further evaluate the potential confounding impact of diabetes, supplementary analyses were conducted including DM diagnosis as a covariate while retaining antidiabetic medication use, given the absence of multicollinearity between these variables. Additionally, robustness analyses were performed by excluding AD dementia to assess whether the association between amyloid species and glucose metabolism was preserved among cognitively normal individuals and MCI participants. These sub-analyses aimed to verify the robustness of the primary findings under varying clinical conditions. As an additional exploratory analysis, continuous tau-PET SUVR (meta-ROI) was used in place of Braak stage as a moderator to further examine the dose-dependent interactions between Aβ biomarkers and tau pathology on glucose metabolism.

Lastly, to complement the primary analyses based on continuous biomarker measures, supplementary GLMs were performed using categorical predictors, specifically MDS-OAβ risk group and Aβ-PET positivity. These models were included as supplementary analyses to provide descriptive context, while acknowledging the limited interpretability due to small sample sizes in stratified subgroups. The distribution of HbA1c values across MDS-OAβ risk groups is illustrated in Supplementary Figure S3, providing a visual overview of the data structure relevant to these analyses. Statistical significance was set at *p* < 0.05 (two-tailed).

## 4 Results

### 4.1 Baseline demographic and clinical data

For biomarker verification, 106 Aβ-PET-negative cognitively normal individuals, 116 Aβ-PET-negative MCI, 101 Aβ-PET-positive MCI, and 32 Aβ-PET-positive dementia patients were included (Supplementary Table S1). Plasma MDS-OAβ levels showed a non-significant trend, increasing from Aβ-PET-negative cognitively normal individuals to Aβ-PET-positive MCI, then decreasing in Aβ-PET-positive dementia (*p* = 0.149, Supplementary Figure S4).

In a separate analysis examining the distributional characteristics of core AD biomarkers across Braak stages, global Aβ-PET and tau-PET SUVR (meta-ROI) values increased progressively across Braak stages (*p* < 0.001), whereas plasma MDS-OAβ levels peaked at Braak stage III/IV but did not differ significantly across stages (*p* = 0.193; Supplementary Fig. S5). Plasma MDS-OAβ levels were not significantly correlated with global Aβ-PET SUVR when including all Braak stages (ρ = 0.029, *p* = 0.757) or when restricting the analysis to participants with Braak stage 0 to IV (ρ = 0.106, *p* = 0.332). A positive correlation was observed between global Aβ-PET SUVR and tau-PET SUVR (ρ = 0.679, *p* < 0.001), whereas plasma MDS-OAβ levels were not significantly correlated with tau-PET SUVR (ρ = 0.109, *p* = 0.249).

Among a subset of 113 participants with available tau-PET, HbA1c, and fasting glucose data, demographic and clinical characteristics are presented in Table 1. The frequency of *APOE* ε4 carriers and Aβ-PET SUVR values was highest in the Aβ-PET-positive dementia group (*p* < 0.001). Tau-PET SUVR (meta-ROI) values and Braak stages progressively increased with disease severity, whereas MMSE and CERAD-K total scores decreased (all *p* < 0.001). Group differences were also observed in the prevalence of DM, antidiabetic medication use, and HbA1c levels (*p* < 0.05). Fasting glucose levels and plasma MDS-OAβ levels did not differ significantly across groups (Table 1).

**Table 1:** Baseline demographic, clinical, and biomarker characteristics across diagnostic groups.

	NC (*N* = 28)	Aβ‑PET (-) MCI (*N* = 24)	Aβ‑PET (+) MCI (*N* = 35)	Aβ‑PET (+) dementia (*N* = 26)	*P* value
Age (years)	73.0 (7.5)	75.5 (6.7)	75.3 (6.9)	74.9 (7.5)	0.551
Sex (female)	20 (71.4 %)	18 (75.0 %)	27 (77.1 %)	18 (69.2 %)	0.903
Years of education	9.7 (5.3)	11.1 (4.6)	10.4 (4.7)	11.1 (4.7)	0.676
*APOE* ε4 carrier status (carrier)	8 (28.6 %)	4 (16.7 %)	14 (40.0 %)	19 (73.1 %)	< 0.001
DM	4 (14.3 %)	11 (45.8 %)	5 (14.3 %)	11 (42.3 %)	0.006
Antidiabetic medication	3 (10.7 %)	9 (37.5 %)	4 (11.4 %)	9 (34.6 %)	0.018
HbA1c	6.0 (0.6)	6.4 (1.0)	5.8 (0.8)	6.3 (0.7)	0.025
Fasting blood glucose levels (mg/dl)	118.0 (38.8)	124.9 (39.4)	111.1 (27.8)	135.3 (49.0)	0.107
Plasma MDS-OAβ level (ng/ml)	0.61 (0.21)	0.61 (0.31)	0.60 (0.29)	0.63 (0.22)	0.966
Plasma MDS-OAβ risk					0.781
Low	21 (75.0 %)	16.0 (66.7 %)	25.0 (71.4 %)	17 (65.4 %)	
Intermediate	5 (17.9 %)	5.0 (20.8 %)	6.0 (17.1 %)	8 (30.8 %)	
High	2 (7.1 %)	3.0 (12.5 %)	4.0 (11.4 %)	1 (3.8 %)	
Global Aβ-PET SUVR	0.59 (0.21)	0.47 (0.08)	0.84 (0.12)	0.85 (0.09)	< 0.001
Aβ-PET positivity	9 (32.1 %)	0 (0.0 %)	35 (100.0 %)	26 (100.0 %)	< 0.001
Tau-PET SUVR (meta-ROI)	1.19 (0.13)	1.22 (0.21)	1.59 (0.33)	1.90 (0.34)	< 0.001
Braak stage					< 0.001
Braak 0	22 (78.6 %)	19 (79.2 %)	10 (28.6 %)	2 (7.7 %)	
Braak I	5 (17.9 %)	3 (12.5 %)	5 (14.3 %)	0 (0.0 %)	
Braak III/IV	1 (3.6 %)	2.0 (8.3 %)	10 (28.6 %)	7 (26.9 %)	
Braak V/VI	0 (0.0 %)	0 (0.0 %)	10 (28.6 %)	17 (65.4 %)	
MMSE	26.9 (1.8)	23.6 (3.8)	22.5 (3.7)	17.5 (4.1)	< 0.001
CERAD-K total score	71.1 (8.4)	53.9 (9.6)	51.7 (12.5)	33.5 (8.4)	< 0.001

**Note.** Values are presented as mean ± SD for continuous variables and n (%) for categorical variables. **Abbreviations.** MDS-OAβ, Multimer Detection System oligomeric amyloid-beta; SUVR, standardized uptake value ratio; MMSE, Mini-Mental State Examination; CERAD-K, Korean version of the Consortium to Establish a Registry for Alzheimer’s Disease.

### 4.2 Nonlinear associations between plasma MDS-OAβ and glycemic indices across braak stages

**Fig. 1:**
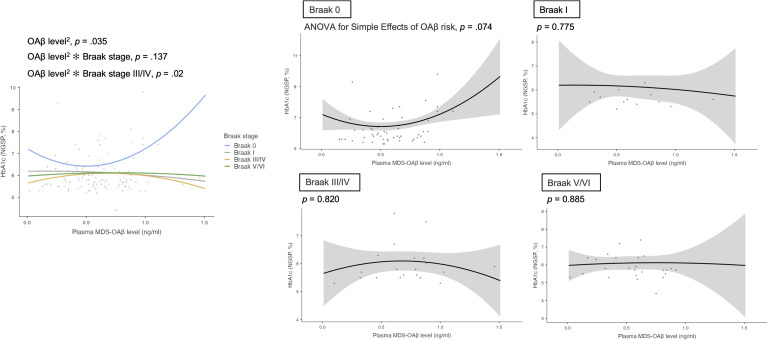
Tauopathy-specific effects of plasma MDS-OAβ levels on HbA1c, incorporating quadratic modeling. **Note.** Analyses adjusted for age, sex, education, *APOE* ε4 carrier status, antidiabetic medication use, and global CDR score (see Methods for details).

**Fig. 2:**
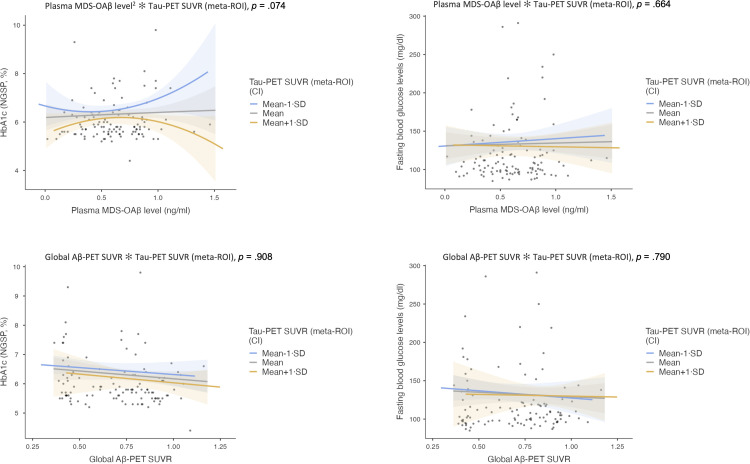
Tauopathy-modulated effects of continuous core AD biomarkers on glycemic indices. **Note.** Generalized linear models were used to examine the interaction effects between continuous AD biomarkers (plasma MDS-OAβ levels or global Aβ-PET SUVR) and Tau-PET SUVR (meta-ROI) on glycemic indices. Models were adjusted for age, sex, education, *APOE* ε4 carrier status, antidiabetic medication use, and global CDR score.

Although the global model comparison did not indicate a statistically significant improvement in overall fit [LRT χ²(4) = 6.44, *p* = 0.169; ΔAIC = +1.56, ΔBIC = +12.47], GLMs incorporating quadratic terms for plasma MDS-OAβ revealed a significant stage-specific nonlinear interaction affecting HbA1c levels. As shown in Table 2 and Fig. 1, the squared plasma MDS-OAβ level exhibited a significant interaction with Braak stage III/IV on HbA1c (β = –4.191, 95 % CI: –7.714 to –0.669, *p* = 0.020), indicating a stage-specific nonlinear association. The corresponding interaction terms at Braak stage I (β = –3.430, *p* = 0.147) and V/VI (β = –3.453, *p* = 0.119) did not reach statistical significance. Full coefficient tables for the quadratic specification are provided in Supplementary Table S2. In a separate model using continuous tau-SUVR as a moderator (Fig. 2), a similar pattern was observed, although the interaction did not reach significance (β = –0.231, *p* = 0.074).

**Table 2:** Interaction effects of MDS-OAβ levels or Aβ-PET SUVR with Braak stage on glycemic indices.

Glycemic indices	Interaction effects	Estimate (β)	95 % CI	*P*-value
HbA1c	MDS-OAβ level^2^ × Braak I	−3.430	(−8.070, 1.209)	0.147
	MDS-OAβ level^2^ × Braak III/IV	−4.191	(−7.714, −0.669)	**0.020**
	MDS-OAβ level^2^ × Braak V/VI	−3.453	(−7.795, 0.888)	0.119
	Aβ-PET SUVR × Braak I	1.747	(−0.753, 4.248)	0.171
	Aβ-PET SUVR × Braak III/IV	0.289	(−2.354, 2.931)	0.830
	Aβ-PET SUVR × Braak V/VI	0.036	(−2.363, 2.434)	0.977
Fasting blood glucose	MDS-OAβ level × Braak I	−18.139	(−93.464, 57.185)	0.637
	MDS-OAβ level × Braak III/IV	−15.119	(−77.254, 47.016)	0.633
	MDS-OAβ level × Braak V/VI	0.345	(−63.880, 64.570)	0.992
	Aβ-PET SUVR × Braak I	4.874	(−124.422, 134.170)	0.941
	Aβ-PET SUVR × Braak III/IV	−71.667	(−208.311, 64.977)	0.304
	Aβ-PET SUVR × Braak V/VI	1.204	(−122.806, 125.214)	0.985

**Note.** Generalized linear models assessed interaction effects between continuous MDS-OAβ levels (or Aβ-PET SUVR) and categorical Braak stage on glycemic indices, adjusting for age, sex, education, *APOE* ε4 status, antidiabetic medication use, and global CDR score. Braak stage 0 served as the reference. Estimates reflect differences in the association slopes at each Braak stage relative to the reference.

### 4.3 Associations between global Aβ-PET SUVR and glycemic indices across tau pathology severity

No significant interactions were observed between global Aβ-PET SUVR and Braak stage in relation to HbA1c or fasting glucose (Table 2). Interaction terms between Aβ-PET SUVR and Braak stage III/IV yielded β = 0.289 (95 % CI: –2.354 to 2.931, *p* = 0.830) for HbA1c and β = –71.667 (95 % CI: –208.311 to 64.977, *p* = 0.304) for fasting glucose. No interaction effects reached statistical significance across tau pathology severity (Table 2). In the model using tau-PET SUVR as a continuous moderator (Fig. 2), interaction effects remained non-significant (all *p* > 0.3), and no evidence of nonlinear associations was observed.

### 4.4 Robustness analyses

Results were consistent in sensitivity analyses that included diabetes diagnosis as an additional covariate (Supplementary Table S3) and excluded participants with dementia (Supplementary Table S4). The interaction between the squared plasma MDS-OAβ level and Braak stage III/IV on HbA1c remained statistically significant in both models (*p* = 0.019 and *p* = 0.024, respectively). Interaction terms at other stages remained non-significant (*p* > 0.05), supporting the robustness of the observed association pattern. To further explore whether the observed stage-dependent associations might be attributable to differences in the variability of plasma MDS-OAβ levels across tauopathy stages, we compared distributional characteristics of plasma MDS-OAβ levels between advanced and lower Braak stages. The standard deviation (0.284 vs. 0.244) and interquartile range (0.395 vs. 0.348) were comparable, suggesting that variance differences are unlikely to explain the observed interaction effects.

### 4.5 Secondary analyses based on categorical risk classification

To complement the continuous biomarker models, secondary analyses were performed using categorical predictors: MDS-OAβ risk group and Aβ-PET positivity. As shown in Supplementary Table S5 and Figure S6, high MDS-OAβ risk was associated with significantly lower HbA1c levels at Braak stage I (β = –2.010, 95 % CI: –3.087 to –0.933, *p* < 0.001) and III/IV (β = –1.785, 95 % CI: –2.739 to –0.831, *p* < 0.001) compared to low-risk participants. No significant interaction was observed at Braak stage V/VI (*p* = 0.208) or in fasting glucose models (*p* > 0.3 for all terms). Aβ-PET positivity showed no significant interaction effects across Braak stages (*p* > 0.3).

## 5 Discussion

In this study, we investigated how OAβ and amyloid plaques are differentially related to systemic glucose metabolism, with attention to the moderating role of tau pathology. We found that OAβ burden was associated with long-term glucose dysregulation (HbA1c), particularly before extensive neurofibrillary tangle accumulation, whereas no significant effects were observed for short-term regulation (fasting glucose). Amyloid plaque burden did not show metabolic interactions with tau pathology, underscoring a more specific role of OAβ in systemic glucose dysfunction.

In our cohort, amyloid-PET and tau-PET positivity, as well as diabetes prevalence, increased progressively across cognitive stages from normal cognition to MCI and Aβ-PET–positive dementia. This trajectory mirrors clinical observations that metabolic impairment often co-occurs with advancing AD pathology. Plasma MDS-OAβ levels did not differ significantly between groups but followed a characteristic course of early elevation and later decline [13]. Notably, levels peaked at Braak III/IV and declined at Braak V/VI, suggesting a shift from soluble oligomers to fibrillar deposits with advancing tau pathology. For glycemic indices, fasting glucose and HbA1c rose from MCI to dementia, but within MCI, levels were lower in Aβ-PET–positive than in Aβ-PET–negative individuals. These findings are consistent with epidemiological data linking poor glycemic control to dementia risk in diabetes [[39], [40], [41]] and underscore heterogeneity across cognitive stages and amyloid status.

Nonlinear modeling showed that the quadratic OAβ–HbA1c association was significant only at Braak III/IV relative to Braak 0, with no significant associations at Braak I or V/VI. This stage-specific effect reflects the non-monotonic OAβ trajectory, rising from Braak 0, peaking at III/IV, and declining at V/VI. Consequently, the contrast in HbA1c levels by OAβ burden was maximized at Braak III/IV, explaining why statistical significance emerged only at this stage. These results indicate that the interaction was not due to simple linear attenuation across tau stages but rather to the distributional pattern of OAβ across disease progression. The association between OAβ burden and long-term glucose dysregulation was therefore most evident in the absence of advanced tau pathology, rather than reflecting a direct link to tau deposition. This interpretation accords with experimental evidence that OAβ disrupts systemic glucose regulation through impaired insulin signaling, synaptic dysfunction, neuroinflammation, and hypothalamic dysregulation [[6], [7], [8], [9]]. Nevertheless, given the larger sample size at Braak 0, differences in statistical power across stages cannot be excluded and results should be interpreted with caution.

Although experimental studies indicate that OAβ can directly impair peripheral insulin signaling, our association with HbA1c may also reflect indirect mechanisms such as hypothalamic insulin resistance or central regulatory dysfunction [6,7,9]. Clarifying these pathways will require longitudinal and mechanistic studies with dynamic metabolic assessments.

The restriction of the OAβ–HbA1c association to participants without detectable tau highlights a potential modulatory role of tau. Aβ oligomers promote tau phosphorylation, and hyperphosphorylated tau or tau loss of function can impair insulin signaling [14,42,43]. Thus, the metabolic effects of OAβ may diminish as tau pathology progresses, possibly due to Aβ–tau interactions that interfere with OAβ-related pathways [44]. Our null findings at higher Braak stages are consistent with prior studies reporting negative or insignificant associations between diabetes or HbA1c and amyloid [4,45]. Taken together, these results suggest that advancing tau pathology may weaken or obscure amyloid–metabolism associations.

Our findings are consistent with reports that tau pathology assessed by PET is not significantly associated with systemic insulin resistance [19], glucose metabolism [46], or diabetes in meta-analyses [4]. By contrast, several studies have shown stronger associations between soluble tau species in CSF and metabolic dysfunction [17,18]. More recently, fasting glucose measured in early adulthood predicted tau-PET decades later [47]. However, given the variability of glucose and reliance on single-time-point measures in nondemented adults, these findings should be interpreted cautiously. Overall, the metabolic effects of tau may vary by pathological stage and molecular form, consistent with evidence from distinct tau biomarkers reflecting different phases of disease progression.

Tau-PET primarily detects mature tangles of later-stage pathology, whereas soluble tau oligomers implicated in early toxicity remain undetectable by conventional imaging. Experimental data show that soluble tau induces neuronal dysfunction through hyperexcitability, altered hippocampal oscillations, and impaired autophagy-lysosomal and mitochondrial processes [48,49]. Because tau oligomers act before fibrillary aggregation [49,50], reliance on tau-PET alone may underestimate early metabolic effects. In our analysis, continuous tau-PET SUVR did not significantly interact with OAβ burden, whereas Braak stage more clearly moderated the OAβ–HbA1c association. These findings support the utility of Braak staging, as endorsed by the revised NIA–AA criteria [51].

Our results indicate that OAβ burden, but not amyloid plaques, was associated with HbA1c, supporting the hypothesis that soluble OAβ plays a central role in chronic glucose regulation in AD. This aligns with evidence that OAβs are the primary neurotoxic species driving synaptic and metabolic dysfunction, whereas plaques are relatively inert [9,52,53]. Prior studies of plaques and glucose metabolism have yielded inconsistent results. Our findings differ from longitudinal work linking midlife insulin resistance to late-life plaque deposition [21,22], but are consistent with cross-sectional studies reporting no significant plaque–metabolism associations [4,45]. Such discrepancies may reflect differences in metabolic measures, with longitudinal studies emphasizing insulin resistance and cross-sectional work hyperglycemia. Importantly, most earlier studies focused on cognitively normal individuals, limiting variability in amyloid-PET and reducing generalizability. A key strength of our study is the application of the revised NIA–AA criteria [51], enabling assessment of AD pathology and metabolism across a broader spectrum. Nonetheless, larger studies are required to clarify these relationships.

Finally, we found a significant association between OAβ burden and HbA1c but not with fasting glucose. Although both short- and long-term glycemic impairments have been linked to dementia risk [39,54], prior studies have reported inconsistent associations with Aβ [4,55]. Such discrepancies may reflect differences in amyloid-PET methodology, disease stage, and confounding factors such as antidiabetic medication. While further replication is needed, our results suggest that OAβ burden is specifically linked to long-term metabolic stress, pointing to chronic hyperglycemia as a potential therapeutic target.

The cross-sectional design of this study limits causal inference between AD pathology and systemic glucose metabolism. Although experimental data suggest that OAβ can impair insulin signaling and hypothalamic function [6,7,9], our findings cannot establish temporality or mechanistic links. Unmeasured confounders and stage-dependent variability may also have influenced results. Furthermore, systemic dysfunction may precede and contribute to AD pathology, as shown previously [1,2], raising the possibility of a bidirectional relationship. Longitudinal studies with serial metabolic assessments and biomarker profiling will be essential to clarify temporal dynamics. To strengthen future work, such designs should integrate multimodal biomarkers, including tau-PET, fluid-based tau markers, and direct measures of insulin resistance, to comprehensively characterize the sequence of metabolic and pathological changes.

We examined soluble OAβ as a biomarker of systemic dysfunction based on its mechanistic link to glucose dysregulation. The MDS assay measures oligomerization tendency rather than absolute levels or structural features of OAβ, requiring cautious interpretation [56,57]. In addition, we did not directly measure insulin resistance, relying instead on glycemic indices and diabetes status as surrogates. Although clinically validated, these indices may not fully capture systemic insulin sensitivity. Incorporating direct assessments of insulin resistance, such as HOMA-IR or clamp techniques, would help determine whether OAβ contributes through insulin-related pathways rather than downstream metabolic consequences. Future studies combining these approaches with additional AD biomarkers and tau-PET imaging will be critical to validate the observed associations.

The nonlinear OAβ–HbA1c association and tau-stage interactions should also be interpreted cautiously due to limited subgroup sample size. Although stage-specific effects did not consistently reach significance, the overall pattern, combined with model robustness to distributional assumptions and outliers, suggests that the findings are unlikely due to chance alone. The attenuation of OAβ–HbA1c associations at advanced tau stages was not explained by reduced OAβ variance or increased diabetes prevalence, underscoring the complexity of amyloid, tau, and metabolic interactions. Finally, the temporal disconnect between long-term amyloid and tau accumulation and short-term glucose variability may also have contributed to the observed results [58]. Clarifying these mechanisms will require larger longitudinal cohorts with comprehensive biomarker and metabolic assessments.

Our findings demonstrated distinct interaction patterns between OAβ burden and tau pathology in relation to long-term glucose dysregulation, most evident in participants without tau deposition. This underscores the biological relevance of soluble OAβ and highlights the potential role of tau in shaping systemic metabolic alterations in AD. Clarifying these interactions may help identify therapeutic targets and inform the timing of interventions for AD-related metabolic dysfunction.

## Availability of data and materials

The datasets generated or analyzed during the current study are not publicly available due to the Patient Data Management Protocol of Yeouido Saint Mary’s Hospital but are available from the corresponding author upon reasonable request.

## Conflicts of interest

Hyun Kook Lim, Yeong Sim Choe, and Donghyeon Kim are employees of NEUROPHET Inc. The data processing services used in this study were provided by NEUROPHET Inc. to support brain imaging analysis. The authors affirm that the study findings and interpretations are solely those of the authors and were not influenced by the company. All other authors declare no conflicts of interest.

## Declaration of generative AI and AI-assisted technologies in the writing process

During the preparation of this work, the authors used ChatGPT (OpenAI) solely for the purpose of English language editing. After using this tool, the authors carefully reviewed and edited the content as needed and take full responsibility for the content of the publication. No scientific content was generated or substantively modified by the AI beyond language refinement, and the overall contribution of AI-assisted technologies was limited to less than 10 % of the total writing process.

## Funding

This research was supported by the Culture, Sports, and Tourism R&D Program through the Korea Creative Content Agency grant funded by the Ministry of Culture, Sports, and Tourism of Korea in 2023 (R2022020030); the Basic Medical Science Facilitation Program through the Catholic Medical Center of the Catholic University of Korea funded by the Catholic Education Foundation; the Institute of Clinical Medicine Research at Yeouido St. Mary’s Hospital, the Catholic University of Korea; the Basic Science Research Program through the National Research Foundation of Korea (NRF) funded by the Ministry of Education (2022R1I1A1A01053710); and a research fund of Seoul St. Mary’s Hospital, the Catholic University of Korea (ZC23TISI0860). The sponsors had no role in the design and conduct of the study; in the collection, analysis, and interpretation of data; in the preparation of the manuscript; or in the review or approval of the manuscript.

## CRediT authorship contribution statement

**Dong Woo Kang:** Writing – original draft, Visualization, Methodology, Funding acquisition, Formal analysis, Conceptualization. **Suhyung Kim:** Visualization, Methodology, Data curation. **Sunghwan Kim:** Visualization, Methodology, Data curation. **Yoo Hyun Um:** Writing – review & editing, Methodology, Data curation. **Sheng-Min Wang:** Writing – review & editing, Methodology, Investigation. **Seunggyun Ha:** Validation. **Sonya Youngju Park:** Validation. **Seung-Hwan Lee:** Validation. **Yeong Sim Choe:** Software. **Donghyeon Kim:** Software. **Chang Uk Lee:** Supervision, Conceptualization. **Hyun Kook Lim:** Writing – review & editing, Supervision, Project administration, Methodology, Funding acquisition, Conceptualization.

## Declaration of competing interest

The authors declare the following financial interests/personal relationships which may be considered as potential competing interests:

Hyun Kook Lim, Yeong Sim Choe, and Donghyeon Kim are employees of NEUROPHET Inc. The data processing services used in this study were provided by NEUROPHET Inc. to support brain imaging analysis. The authors affirm that the study findings and interpretations are solely those of the authors and were not influenced by the company. All other authors declare no conflicts of interest. If there are other authors, they declare that they have no known competing financial interests or personal relationships that could have appeared to influence the work reported in this paper.

## Appendix Supplementary materials

Image, application 1
