# Supplementary material for: Soluble and plaque amyloid associations with peripheral glucose dysregulation modulated by tau pathology in Alzheimer’s disease
Source: J Prev Alzheimers Dis. 2026 Jan 1;13(2):100459. doi: 10.1016/j.tjpad.2025.100459 (PMC12869051; doi:10.1016/j.tjpad.2025.100459)
Supplement: Supplementary file 1 [file mmc1.pdf]

Additional File 1: Supplementary Tables, and Figures

File format: .docx

Title: Supplementary Materials for "Soluble and Plaque Amyloid Associations with Peripheral Glucose Dysregulation Modulated by Tau Pathology in Alzheimer's Disease"

Description: This file includes supplementary results, additional subgroup and sensitivity analyses, and supporting figures for the main manuscript. It contains four supplementary tables (S1–S5) and six figures (S1–S6) detailing analytic procedures, model outputs, and secondary results.

Table of Contents

1. **Supplementary Tables**
   - **Table S1.** Baseline demographic and clinical characteristics of the total participants
   - **Table S2.** Tauopathy-specific effects of plasma MDS-OAβ levels on HbA1c, incorporating quadratic modeling
   - **Table S3.** Interaction between Aβ measures and Tauopathy on glycemic indices adjusting for diabetes diagnosis
   - **Table S4.** Interaction between Aβ measures and Tauopathy on glycemic indices excluding AD dementia patients
   - **Table S5.** Interaction effects of Aβ oligomerization risk or Aβ-PET positivity with Braak stage on glycemic indices
2. **Supplementary Figures**

- **Figure S1.** Flowchart of Participant Selection and Data Availability
- **Figure S2.** Residual Diagnostics for GLMs Evaluating Associations with HbA1c and Fasting Glucose
- **Figure S3.** Distribution of HbA1c (%) across MDS-OAβ Risk Groups
- **Figure S4.** MDS-OAß level according to disease stage and Aβ-PET positivity
- **Figure S5.** Group-wise and correlational analysis of AD core biomarkers across Braak stages
- **Figure S6.** Interaction effects of Aβ oligomerization risk and Aβ-PET positivity with Tau Braak stage on glucose metabolism

Supplementary Methods

Glycemic Biomarker Assessment

Fasting blood samples were collected after a minimum of 8 hours of overnight fasting. Plasma glucose levels were measured using the enzymatic hexokinase method (1), which is the recommended standard for clinical laboratory testing due to its high specificity and reproducibility. Hemoglobin A1c (HbA1c) levels were analyzed using high-performance liquid chromatography (HPLC)-based assays that are certified by the National Glycohemoglobin Standardization Program (NGSP) (2) and aligned with the Diabetes Control and Complications Trial (DCCT) and International Federation of Clinical Chemistry and Laboratory Medicine (IFCC) standards. These methods were chosen to ensure consistency with internationally accepted diagnostic criteria for diabetes mellitus. The assays were conducted in a certified laboratory with routine quality control procedures in place to maintain analytical validity.

Neuropsychological evaluation

Cognitive status was assessed using neuropsychological testing at Yeouido St. Mary's Hospital, The Catholic University of Korea. All participants underwent cognitive evaluation using the Korean version of the Consortium to Establish a Registry for Alzheimer's Disease (CERAD-K), a standardized battery for the assessment of cognitive function in Alzheimer's disease. The battery included Verbal Fluency (VF), the 15-item Boston Naming Test (BNT), the Korean Mini-Mental State Examination (MMSE-K), Word List Memory (WLM), Word List Recall

(WLR), Word List Recognition (WLRc), Constructional Praxis (CP), and Constructional Recall (CR). Test performance was reviewed by a neuropsychologist to determine the presence of cognitive impairment.

Individual subtest scores ranged as follows: VF (number of animal names in 1 minute), BNT (0–15), MMSE-K (0–30), WLM (0–30), WLR (0–10), WLRc (0–10), CP (0–11), and CR (0–11). A comprehensive CERAD-K score (range: 0–100) was computed by summing all subdomain scores except MMSE-K and CR. Higher scores in each subdomain reflected better cognitive performance.

APOE genotyping

DNA was isolated from blood using the QIAmp Blood DNA Maxi Kit protocol (Qiagen, Valencia, CA). Genotypes for two APOE SNPs, rs429358 (E*4) and rs7412 (E*2) were determined using TaqMan SNP genotyping assays (Applied Biosystems, Foster City, California).

Supplementary Table S1. Baseline demographic and clinical characteristics of the total participants

	Aβ-PET (-) NC (N=106)	Aβ-PET (+) NC (N=18)	Aβ-PET (-) MCI (N=116)	Aβ-PET (+) MCI (N=101)	Aβ-PET (+) dementia (N=32)	*P* value
Age (mean ± SD, years)	71.5 (7.6)	76.3 (6.5)	77.4 (6.2)	74.3 (7.3)	73.8 (8.5)	< 0.001
Sex (female, %)	74 (69.8%)	14 (77.8%)	87 (75.0%)	77 (76.2%)	21 (65.6%)	0.660
Years of education (mean ± SD)	11.4 (4.8)	9.1 (5.1)	9.8 (5.2)	10.4 (5.5)	10.3 (5.0)	0.176
APOE ε4 carrier status (carrier, %)	23 (21.7%)	7 (38.9%)	29 (25.0%)	56 (55.4%)	21 (65.6%)	< 0.001
Plasma MDS-OAβ level (mean ± SD, ng/ml)	0.61 (0.25)	0.63 (0.22)	0.64 (0.28)	0.69 (0.27)	0.60 (0.23)	0.243
Global [^{18}F] Flutemetamol $SUVR_{PONS}$ (mean ± SD)	0.44 (0.07)	0.75 (0.08)	0.45 (0.08)	0.74 (0.07)	0.75 (0.08)	< 0.001
CERAD-K Battery (mean ± SD)						
- VF	15.6 (4.7)	14.3 (2.5)	9.9 (3.7)	10.8 (4.3)	6.4 (3.4)	< 0.001
- BNT	12.7 (1.7)	11.7 (2.4)	10.3 (2.6)	10.1 (3.1)	7.2 (3.3)	< 0.001
- MMSE	27.3 (2.0)	26.2 (1.8)	22.6 (4.3)	22.2 (4.4)	16.7 (3.9)	< 0.001
- WLM	18.8 (3.5)	18.1 (2.8)	13.5 (3.6)	13.1 (3.8)	8.4 (2.6)	< 0.001
- CP	10.4 (1.0)	10.1 (1.0)	9.3 (1.6)	9.2 (1.9)	7.7 (2.8)	< 0.001
- WLR	6.4 (1.6)	6.0 (1.7)	2.9 (1.8)	2.2 (1.7)	0.5 (0.8)	< 0.001
- WLRc	9.2 (0.9)	8.9 (1.2)	6.3 (2.5)	5.7 (2.5)	2.3 (2.2)	< 0.001
- CR	7.1 (2.8)	6.1 (2.8)	2.8 (2.8)	2.4 (2.4)	0.3 (1.0)	< 0.001
- CERAD total score	71.8 (11.2)	69.1 (8.2)	53.8 (21.5)	51.1 (12.6)	32.4 (8.2)	< 0.001

Note. The data are presented as “mean± SD” format for continuous variables and “counts (proportion in percentage)” format for categorical variables. Abbreviations: $SUVR_{PONS}$, standardized uptake value ratio of [^{18}F] Flutemetamol, using the pons as a reference region; CERAD-K, Korean version of Consortium to Establish a Registry for Alzheimer’s Disease; VF, verbal fluency; BNT, Boston Naming Test; MMSE, the Korean version of the Mini-Mental Status Examination; WLM, Word List Memory; CP, Constructional Praxis; WLR, Word List Recall; WLRc, Word List Recognition; CR, Constructional Recall; CERAD total score, composite score summing scores of the CERAD-K VF, BNT, WLM, CP, WLR, and WLRc domains.

Supplementary Table S2. Tauopathy-specific effects of plasma MDS-OAβ levels on HbA1c, incorporating quadratic modeling

AD core biomarkers	X^2	Estimate (β) with 95% CI	*p*-value
MDS-OAβ level	3.217	0.688 (-0.06375, 1.43933)	0.073
MDS-OAβ level^2	4.450	3.186 (0.22580, 6.14520)	**0.035**
Braak stage	4.038		0.257
Braak I		-0.317 (-0.79092, 0.15678)	0.190
Braak III/IV		-0.364 (-0.79171, 0.06383)	0.095
Braak V/VI		-0.343 (-0.79912, 0.11380)	0.141
MDS-OAβ level × Braak stage	1.679		0.642
MDS-OAβ level × Braak I		-0.920 (-2.68436, 0.84531)	0.307
MDS-OAβ level × Braak III/IV		-0.559 (-1.87102, 0.75285)	0.404
MDS-OAβ level × Braak V/VI		-0.611 (-1.92197, 0.70069)	0.361
MDS-OAβ level^2 × Braak stage	5.525		0.137
MDS-OAβ level^2 × Braak I		-3.4304 (-8.07022, 1.20932)	0.147
MDS-OAβ level^2 × Braak III/IV		-4.1913 (-7.71380, -0.66872)	**0.020**
MDS-OAβ level^2 × Braak V/VI		-3.4533 (-7.79472, 0.88813)	0.119

Note: This table presents detailed estimates of the interaction between plasma MDS-OAβ levels (including a quadratic term) and Braak stage on HbA1c, extending the summary interaction results shown in Table 2.

Supplementary Table S3. Interaction between Aβ measures and Tauopathy on glycemic indices adjusting for diabetes diagnosis

Glycemic indices	Interaction effects	Estimate (β)	95% CI	*P*-value
HbA1c	MDS-OAβ level^2 × Braak I	-4.114	(-8.265, 0.037)	0.052
	MDS-OAβ level^2 × Braak III/IV	-3.782	(-6.932, -0.633)	**0.019**
	MDS-OAβ level^2 × Braak V/VI	-2.910	(-6.791, 0.973)	0.142
	Aβ-PET SUVR × Braak I	1.238	(-1.074, 3.551)	0.294
	Aβ-PET SUVR × Braak III/IV	-0.037	(-2.474, 2.399)	0.976
	Aβ-PET SUVR × Braak V/VI	-0.776	(-3.013, 1.462)	0.497
Fasting blood glucose	MDS-OAβ level × Braak I	-29.178	(-104.584, 46.228)	0.448
	MDS-OAβ level × Braak III/IV	-19.735	(-81.359, 41.889)	0.530
	MDS-OAβ level × Braak V/VI	4.937	(-58.745, 68.619)	0.879
	Aβ-PET SUVR × Braak I	-5.811	(-134.658, 123.036)	0.930
	Aβ-PET SUVR × Braak III/IV	-78.519	(-214.265, 57.226)	0.257
	Aβ-PET SUVR × Braak V/VI	-15.822	(-140.467, 108.823)	0.804

Supplementary Table S4. Interaction between Aβ measures and Tauopathy on glycemic indices excluding AD dementia patients

Glycemic indices	Interaction effects	Estimate (β)	95% CI	*P*-value
HbA1c	MDS-OAβ level^2 × Braak I	-3.914	(-8.752, 0.924)	0.113
	MDS-OAβ level^2 × Braak III/IV	-4.275	(-7.987, -0.563)	**0.024**
	MDS-OAβ level^2 × Braak V/VI	-5.302	(-12.348, 1.743	0.140
	Aβ-PET SUVR × Braak I	1.436	(-1.209, 4.081)	0.287
	Aβ-PET SUVR × Braak III/IV	1.201	(-1.827, 4.229)	0.437
	Aβ-PET SUVR × Braak V/VI	-0.004	(-3.050, 3.043)	0.998
Fasting blood glucose	MDS-OAβ level × Braak I	-4.536	(-73.129, 64.058)	0.897
	MDS-OAβ level × Braak III/IV	-32.825	(-90.276, 24.626)	0.263
	MDS-OAβ level × Braak V/VI	-5.111	(-82.400, 72.178)	0.897
	Aβ-PET SUVR × Braak I	3.547	(-116.123, 123.218)	0.954
	Aβ-PET SUVR × Braak III/IV	24.497	(-112.477, 161.470)	0.726
	Aβ-PET SUVR × Braak V/VI	25.656	(-112.171, 163.483)	0.715

Supplementary Table S5. Interaction effects of Aβ oligomerization risk or Aβ-PET positivity with Braak stage on glycemic indices

(A) Aβ Oligomerization Risk

Glycemic indices	Interaction effects	Estimate (β)	95% CI	*P*-value
HbA1c	Intermediate risk × Braak I	0.001	(-0.966, 0.986)	0.984
	High risk × Braak I	-2.010	(-3.087, -0.933)	**<.001**
	Intermediate risk × Braak III/IV	0.201	(-0.484, 0.885)	0.566
	High risk × Braak III/IV	-1.785	(-2.739, -0.831)	**<.001**
	Intermediate risk × Braak V/VI	-0.113	(-0.780, 0.555)	0.741
	High risk × Braak V/VI	-0.861	(-2.201, 0.479)	0.208
Fasting blood glucose	Intermediate risk × Braak I	7.688	(-48.807, 64.183)	0.790
	High risk × Braak I	-29.169	(-91.501, 33.164)	0.359
	Intermediate risk × Braak III/IV	10.359	(-29.241, 49.959)	0.608
	High risk × Braak III/IV	-17.681	(-72.902, 37.539)	0.530
	Intermediate risk × Braak V/VI	-13.950	(-52.574, 24.674)	0.479
	High risk × Braak V/VI	-0.246	(-77.776, 77.283)	0.995

(B) Aβ-PET positivity

Glycemic indices	Interaction effects	Estimate (β)	95% CI	*P*-value
HbA1c	Aβ-PET positivity × Braak stage I	0.506	(-0.569, 1.807)	0.301
	Aβ-PET positivity × Braak stage III/IV	0.212	(-0.971, 1.490)	0.675
Fasting blood glucose	Aβ-PET positivity × Braak stage I	-3.813	(3.39e-24, 1.44e+20)	0.880
	Aβ-PET positivity × Braak stage III/IV	-0.823	(1.11e-23, 1.74e+22)	0.975

Note. Generalized linear models were used to assess the interaction between Aβ oligomerization risk (or Aβ-PET positivity) and Braak stage on HbA1c and Fasting blood glucose levels, adjusting for age, sex, education, *APOE* ε4 carrier status, antidiabetic medication use, and global CDR score. Reference categories were set as Low Aβ oligomerization risk (or Aβ-PET negativity) and Braak stage 0, respectively. Interaction terms represent pairwise comparisons across risk levels and Braak stages relative to these references. The interaction term for Braak stage V/VI × Aβ-PET negativity could not be estimated due to the absence of Aβ-PET-negative individuals in this stage.

Supplementary Figure S1. Flowchart of Participant Selection and Data Availability

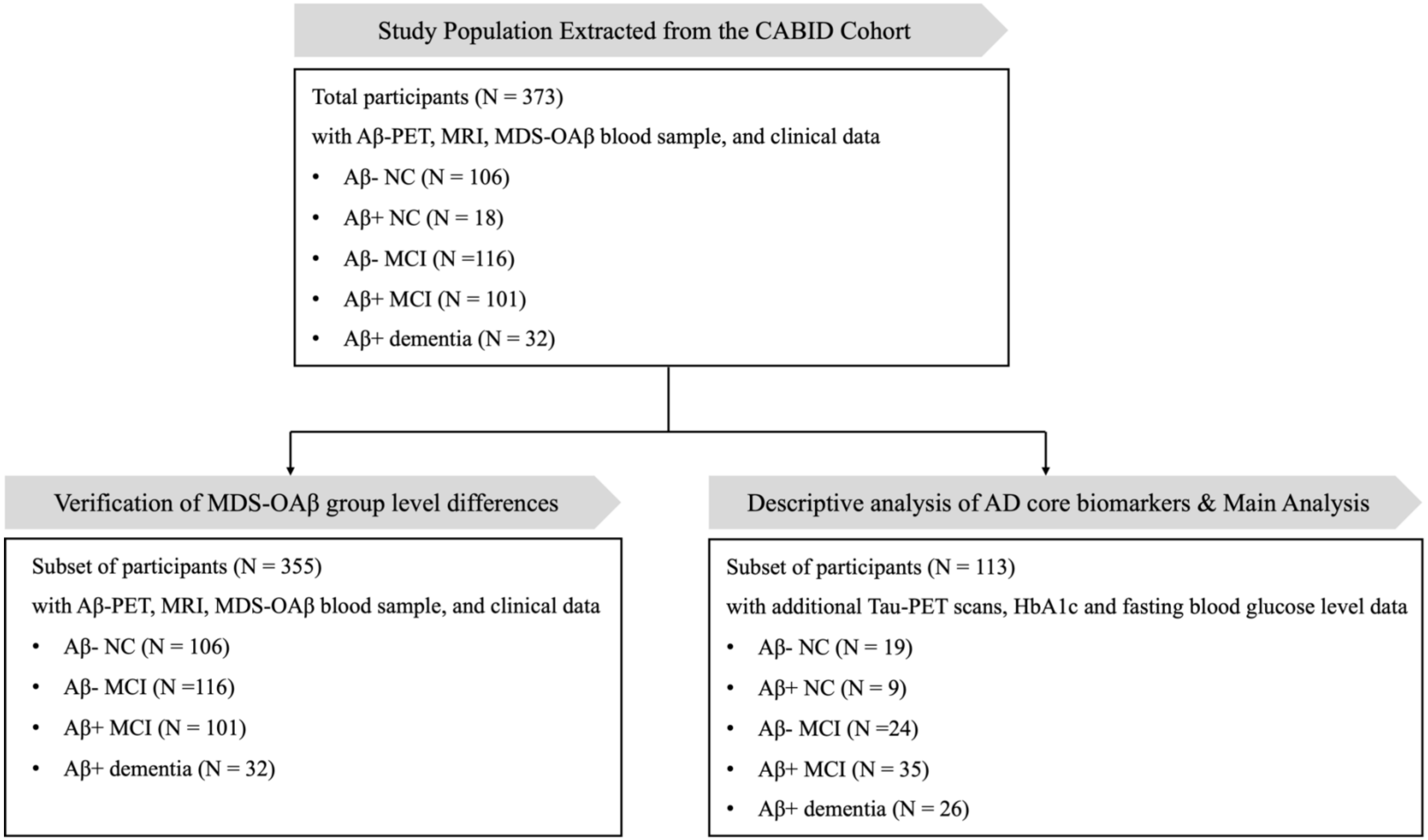

Supplementary Figure S2. Residual Diagnostics for GLMs Evaluating Associations with HbA1c and Fasting Glucose

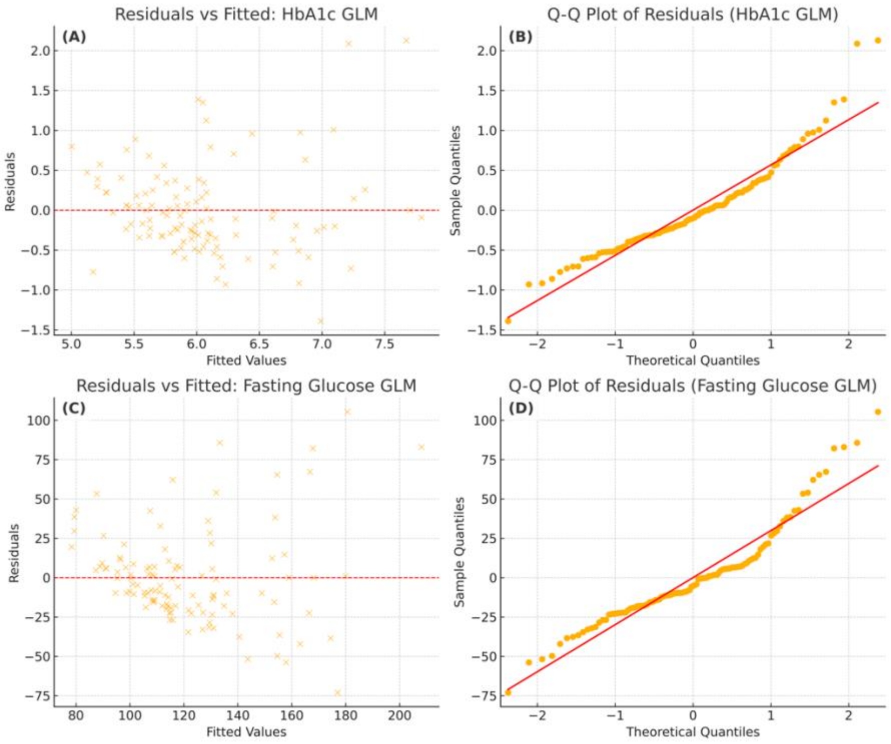

Note. (A) Residuals versus fitted values for the HbA1c GLM, showing no major pattern of heteroskedasticity. (B) Q-Q plot of residuals for the HbA1c GLM, confirming approximate normality with minor deviations at the tails. (C) Residuals versus fitted values for the fasting glucose GLM, demonstrating acceptable scatter without a clear pattern. (D) Q-Q plot of residuals for the fasting glucose GLM, indicating that the residuals align well with the theoretical quantiles, supporting the robustness of the GLMs with HC3 robust standard errors despite slight deviations at the extremes.

Supplementary Figure S3. Distribution of HbA1c (%) across MDS-OAβ Risk Groups

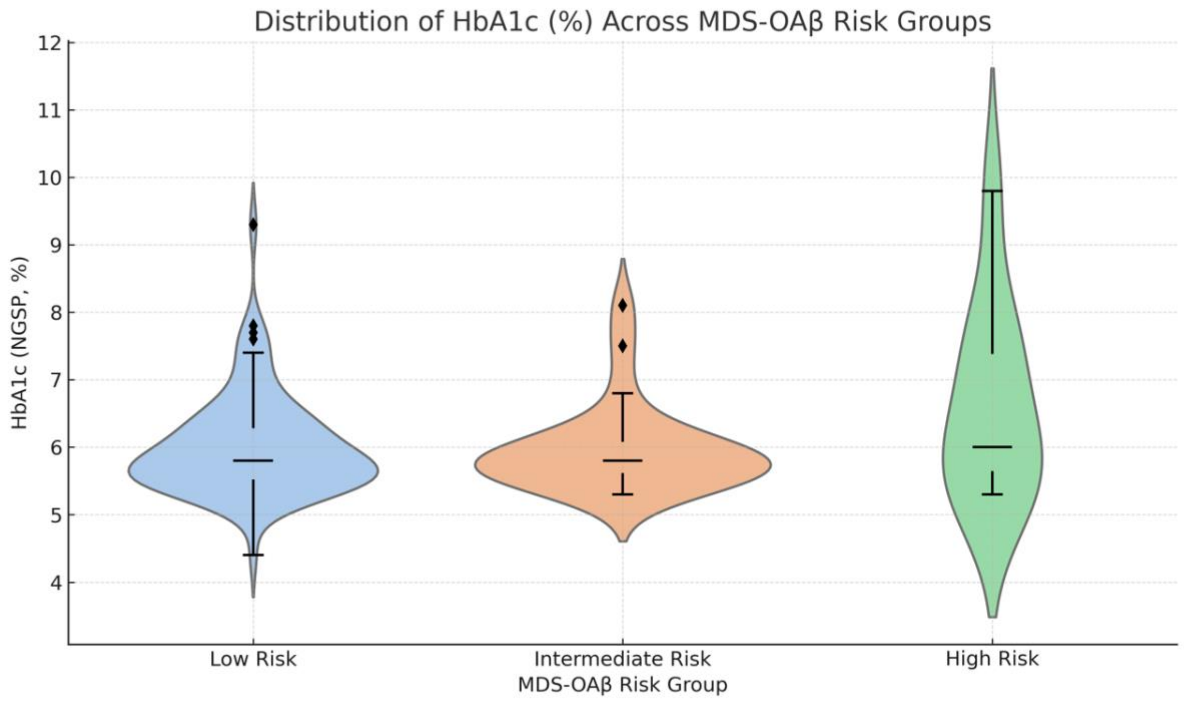

Note. Violin plots with overlaid boxplots illustrate the distribution of HbA1c levels across plasma MDS-OAβ risk groups. The high-risk group demonstrates a right-skewed distribution with elevated HbA1c values, while low- and intermediate-risk groups show narrower distributions. Although deviations from normality were observed in the low- and intermediate-risk groups, no extreme outliers were detected. This supports the robustness of the statistical findings despite differences in sample size.

Supplementary Figure S4. MDS-OAß level according to disease stage and Aβ-PET positivity

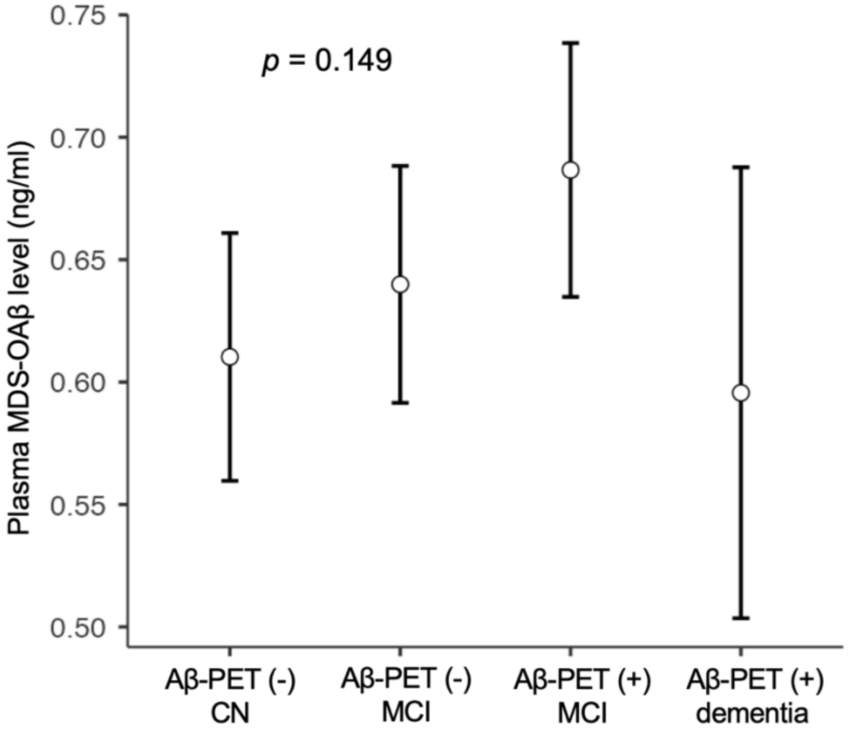

Note. Analysis of variance (ANOVA), Abbreviations: CN: cognitive normal older adults; MCI: Mild cognitive impairment

Supplementary Figure S5. Group-wise and correlational analysis of AD core biomarkers across Braak stages

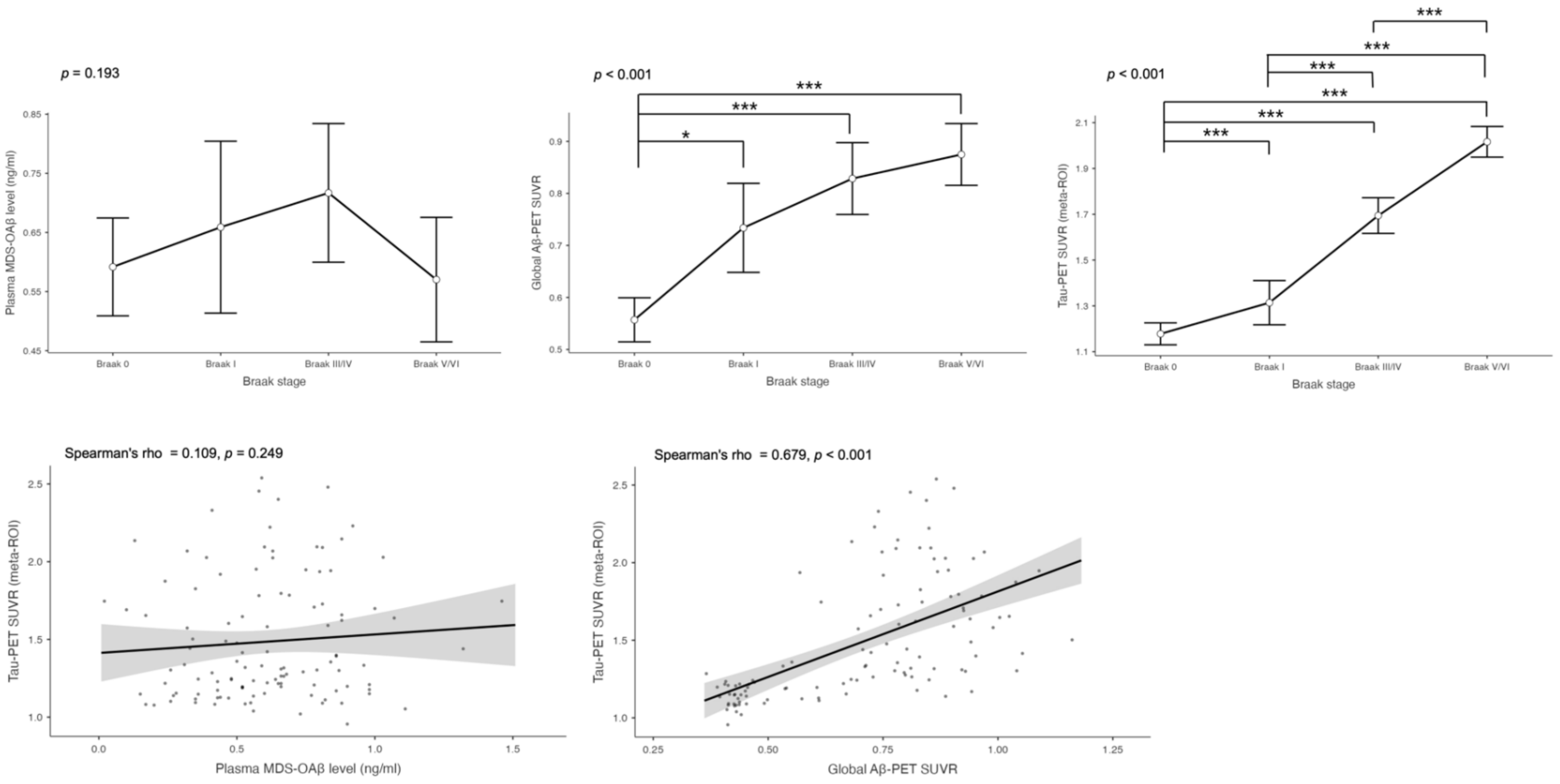

Note. Plasma MDS-OAβ, global Aβ-PET SUVR, and Tau-PET SUVR (meta-ROI) were compared across Braak stages using one-way ANOVA with Bonferroni-corrected post-hoc tests. Spearman correlation analyses were conducted to assess the associations of plasma MDS-OAβ and global Aβ-PET SUVR with Tau-PET SUVR (meta-ROI). Bonferroni-adjusted significance: * $p < .05$, ** $p < .01$, *** $p < .001$.

Supplementary Figure S6. Interaction effects of Aβ oligomerization risk and Aβ-PET positivity with Tau Braak stage on glucose metabolism

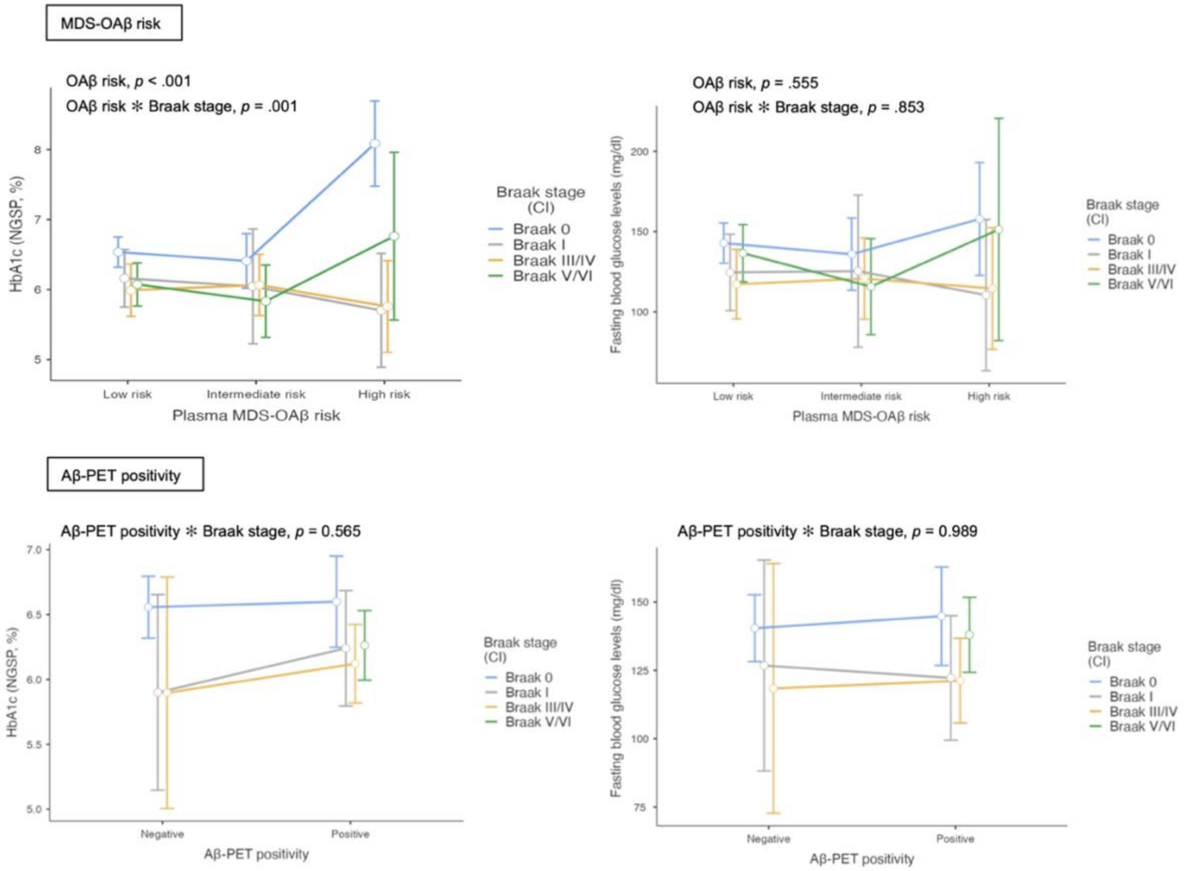

Note. Analyses adjusted for age, sex, education, *APOE* ε4 carrier status, antidiabetic medication use, and global CDR score (see Methods for details).

References

1. Sonagra AD, Zubair M, Motiani A. Hexokinase method. StatPearls [Internet]. StatPearls Publishing,
2. Little RR, Rohlfing C, Sacks DB. The national glycohemoglobin standardization program: over 20 years of improving hemoglobin A1c measurement. Clinical chemistry. 2019;65(7):839-848. https://doi.org/10.1373/clinchem.2018.296962
